# Usability Testing of Virtual Reality Applications—The Pilot Study

**DOI:** 10.3390/s22041342

**Published:** 2022-02-10

**Authors:** Dorota Kamińska, Grzegorz Zwoliński, Anna Laska-Leśniewicz

**Affiliations:** Institute of Mechatronics and Information Systems, Lodz University of Technology, 90-924 Lodz, Poland; grzegorz.zwolinski@p.lodz.pl (G.Z.); anna.laska@p.lodz.pl (A.L.-L.)

**Keywords:** usability, UX, testing, validation, virtual reality, human-computer interaction

## Abstract

The need for objective data-driven usability testing of VR applications is becoming more tangible with the rapid development of numerous VR applications and their increased accessibility. Traditional methods of testing are too time and resource consuming and might provide results that are highly subjective. Thus, the aim of this article is to explore the possibility of automation of usability testing of VR applications by using objective features such as HMD built-in head and hands tracking, EEG sensor, video recording, and other measurable parameters in addition to automated analysis of subjective data provided in questionnaires. For this purpose, a simple VR application was created which comprised relatively easy tasks that did not generate stress for the users. Fourteen volunteers took part in the study and their signals were monitored to acquire objective automated data. At the same time the observer was taking notes of subjects’ behaviour, and their subjective opinions about the experience were recorded in a post-experiment questionnaire. The results acquired from signal monitoring and questionnaires were juxtaposed with observation and post-interview results to confirm the validity and efficacy of automated usability testing. The results were very promising, proving that automated usability testing of VR applications is potentially achievable.

## 1. Introduction

According to ISO 9241 standard of 1998 with additional details of ISO/IEC 25010, usability is the effectiveness, efficiency, and satisfaction of specified users who achieve specified goals in a particular context of use. In this case, effectiveness means the accuracy and completeness with which specified users can achieve those goals [1]. Human Computer Interaction (HCI) and usability have their origins in the 1980s, related to increasing popularity of personal computers among ordinary users, caused by falling prices of computer hardware. To stimulate this trend software design had to be user-friendly, which means intuitive and easy to use by casual users, not only trained technical computer specialists. According to the guru of Web page usability, Jakob Nielsen [2], usability can be defined as a set of 5 elements:

- Learnability: How easy it is for the user to accomplish basic tasks the first time they are dealing with the design.
- Efficiency: How quickly the user can perform tasks once they have learned the design.
- Memorability: How easily the user reestablishes proficiency when they return to the design after a period of not using it.
- Errors: How many errors the users make, and how easily they can recover from the errors.
- Satisfaction: How pleasurable it is to use the design.

**Figure 1:**
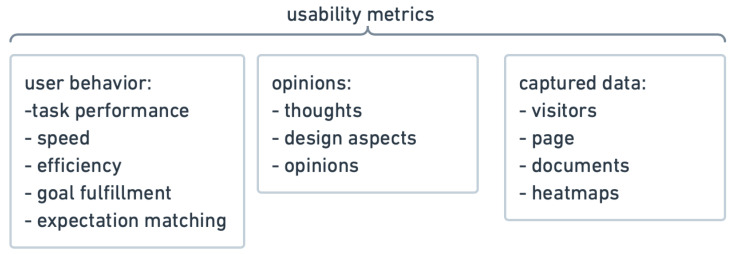
The most commonly used usability testing metrics divided into three categories: user behavior, thoughts and opinions, and captured data (figure based on [12]).

Usability testing is a technique to evaluate a product by testing it on future users, which provides direct input on how they use the system. It aims to check whether the product meets the assumed goals and requirements. Examples of products that most often benefit from usability testing are websites, web applications, computer interfaces, and physical products [3]. There are many methods for improving usability, but the most basic and useful is user testing. Such testing is paramount to the success of the end design of a fully functioning product [4]. There are two types of usability tests: formative and summative user testing. At the early stages of the design and development, the formative method is used to identify and provide solutions to solve interface design issues [5]. Formative testing is an excellent tool to figure out which design features are valuable and which are dispensable. Thus, testers have a significant influence on the further development of the product design. This type of testing is conducted among a small group of users (5–7 is usually enough). The data collected during testing sessions is based on the observation of the quality of the design. Therefore, it is also called qualitative usability testing [6]. The process is iterative [5]. In contrast to formative testing, summative testing is conducted to evaluate the efficacy of the final design, so it is usually done after the product has been launched in the market [7]. It provides an indirect assessment of the design from a larger sample size (the ISO standard takes up to 50 users), usually in a remote form. The usability of the design is measured based on the performance of the testers. The goal is to capture metrics, and the data collected is more about the quantity than the quality of the design. Therefore, it is called quantitative usability testing [8]. As one can easily notice, formative and summative usability testing have significant differences in the study setups, analysis methods, and data outcomes. The most commonly used measures (usability metrics) can be divided into three categories: user behavior (during observation), their thoughts and opinions (surveys, interviews), and captured data (like click path or eye-tracking heat-maps) [9,10,11]. Examples of such measures are presented in Figure 1 and will be described in more detail in the next section.

It should also be emphasized that usability testing protocol strictly depends on evaluated design. Dynamic growth and interest in virtual reality (VR) not only for gaming but also in such areas as medicine [13,14], education [15], business meetings [16,17], and online conferences [18,19] calls for strict and precise usability testing for VR applications.

In contrast to real world face-to-face meetings, the designer of the VR environment has to consider issues such as perception, navigation, exploration, and engagement. They provide the much more significant challenges of a coherent approach to design and understanding the concept of usability in the context of VR, especially as the conventional HCI methods or guidelines are not fully applicable [20]. Therefore, VR designers should strive to maximize intuitive operation, which is easiest to achieve based on real scenarios (commonly known templates for dealing with the natural world) even if the user is placed in a highly unreal world. As it has been highlighted in the previous paragraph, VR design is governed by its own rules and there is still minimal best practice established. Therefore, usability testing plays a crucial role in creating a well-designed VR application. However, only a small amount of work has been done to explore this topic [20]. Most of it is strictly related to a specific application [21], usually using standard approaches such as different system usability scale (SUS) methods [22], post surveys, or interviews [23]. Thus, there is a need for a general walk-through blueprint for evaluating VR user interfaces, especially when it comes to objective data such as bio-signal or behavioral analysis [24]. According to our best knowledge, an approach for usability testing in VR that does not require a continuous presence of the researcher and is scalable has not yet been developed and tested. The question this paper attempts to explore is the possibility of automating usability testing in VR without breaking the immersion and disturbing the subjects, providing objective results. To address the issues mentioned above, this work presents the following contributions:

- We introduce a procedure for usability testing in VR. The procedure includes an automatic analysis of the objective data (e.g., EEG and speech signal analysis, head and hand tracking, speed of task performance) extracted during typical usability testing, and automatic analysis of subjective data represented by questionnaire answers.
- The introduced procedure was tested on fourteen subjects. The results are juxtaposed with specialists’ observations as well as user subjective opinions (post-interview).
- All devices used for collecting objective data are integrated with the headset, thus there is no need for additional external measuring or data collecting equipment, which can disturb the VR experience and consequently influence the usability test results.
- The presented solution is especially aimed at performing usability tests at scale, eliminating the need for constant supervision and observation, which is resource-consuming or impossible with bigger test groups.

The rest of the paper is organized as follows: Section 2 reviews the related work in the field of usability testing in VR. Section 3 discusses most typical issues VR designers face and best practices for dealing with those issues. In Section 4 the details of the proposed method are described, followed by the experimental results and discussion. Finally, conclusions are presented in Section 6.

## 2. Related Works

Formative and summative assessment have seen their usage in VR applications. Both methods provide feedback for designers on functional improvements and are very enticing when implementing new systems like VR [25]. However, there is limited guidance on selecting and performing usability evaluations for VR interfaces in comparison with support for other digital technologies [26]. In [27], authors presented a detailed literature review with evaluation methods that allow measurement of several emotional phenomena connected with digital games. This section presents recent and most relevant approaches and tools for usability testing divided into two categories: objective metrics (psychological and behavioral) and user-subjective metrics in VR usability testing.

### 2.1. User-Subjective Metrics in VR Usability Testing

The data source for subjective usability testing is the perceived feelings of users taking part in a VR experience. This method is very common among usability researchers due to the relatively simple data acquisition formula, which does not require laboratory conditions, expensive monitoring equipment, or complex biomedical data analysis post-measurement. Such research does not present a high value for so-called early-stage usability testing, and it is mainly used for fully developed products [28]. The undemanding data acquisition formula is generally based on direct or indirect questionnaires. This method allows the conduction of mass studies on relatively large numbers of respondents with minimal effort. Thus, it appears in many publications. In [29] for usability study of the VR educational application, more than 100 students were surveyed, which is completely sufficient from the point of view of statistical confidence. Such results may be of very high cognitive value. However, it is worth mentioning the significant drawbacks of the described method. Of course, the biggest disadvantage is the subjectivity itself. Very often, the user describes their impressions about VR rather than actual experiences [30]. Moreover, respondents tend to distort the answers, especially since they complete the questionnaire after the entire VR experience [31]. Thus, the actual feedback may be blurred and not reflected in the answers. Another aspect is the ability to eliminate unreliable results completed with low levels of engagement and to select meaningful respondents [28].

Preparing an efficient questionnaire that will capture valuable usability information, including follow-up questions, while not being too long and tedious, is a complex procedure. Thus, very often, ready-made questionnaires and scales are used. For example, in [32], the authors used Game Experience Questionnaire [33] as well as System Usability Scale [34] to investigate users’ impressions and opinions about the experience while using virtual rehabilitation in upper limbs. Additionally, they used a 0–10 Borg’s scale [35] to quantify perceived fatigue levels. The results showed that a VR immersive experience produced lower fatigue and a more enjoyable and positive experience than a non-immersive one, performed in a controlled experiment with 24 healthy samples.

Very often, specialists in particular areas are involved in creating a proper questionnaire and conducting the survey with final users. For example, in [36] the authors evaluated several examples from their practice, including end-users of VR application for identifying expected benefits and challenges in the context of VR rehabilitation. Based on self-reports and observer-rated instruments gathered during 107 VR-based sessions with 34 participants who underwent a stroke, they proved that end-user involvement significantly improved the user interface (UI) quality, and as a result the needs of the clients and therapists were met. The knowledge and skills of therapists and the input from the end-users provided the information about issues of balancing the technical requirements with the goals of the practice, setting, and the needs of the final users.

To sum up, it has to be emphasized that even the simplest form of usability testing is mandatory in VR system implementation. This process allows designers to confront their vision of the product with expectations of end users and determine if the interaction and the virtual environment itself is transparent and intuitive [37], consistent with perception, knowledge, and previous experiences. However, the success of usability testing is determined mainly by the content of the questionnaire and selected samples. Despite the many imperfections of subjective tests, they still remain the primary tool for measuring the usability of the product.

### 2.2. Objective Metrics in VR Usability Testing

Undoubtedly objective metrics providing solid, measurable evidence are much more valuable for UX researchers. Such metrics should not be based on users’ personal opinions. The best practice for obtaining objective metrics is to perform A/B, A/B/n, or multi-variant tests. Thus, designers should create different versions of interaction to test which of the proposed solutions are the most suitable. Obviously, the very idea of what is more suitable is not a formed standard. For example, VR solutions for the entertainment industry require broadly understood user satisfaction [38], while in the case of training/educational applications, the educational quality should be considered. The latter is critical for VR medical systems, e.g., patient treatment, rehabilitation, and medical professional training [26]. For example, in [32] the authors used gamification features such as awarded points, received shots, and successful attacks as variables that reflected users’ performance. What is more, they analyzed electromyogram to detect user fatigue while taking particular tasks. The results were juxtaposed with perceived levels of fatigue, indicating compliance.

Taking into account the above, subjective and objective methods can be considered complementary to each other. Well-formulated subjective usability questionnaires are essential in setting UI courses since they are based on user feedback, while objective analysis confirms UI evaluation. Such an example is presented in [14], where a VR tool to relieve stress was evaluated by 28 office workers whose physiological indicators (heart rate, galvanic skin response, and muscle response) were monitored during the whole session. The obtained results were compared with subjectively perceived stress based on several different questionnaires investigating the users stress level and mood. Objective usability testing methods suffer from major logistical issues [39]. Often, organizing such tests must be preceded by a cumbersome preparatory process. Typically, this type of testing is performed in a laboratory or semi-laboratory setting. In [40], the authors present a framework to objectively assess the surgical skill and generate formative feedback automatically. VR dentistry training simulation was performed on the same teeth that the laboratory-trained students were trained on. It was evaluated during randomized trials on three different training groups (10 students per group, 30 in total): VR training without feedback, formative VR training, and formative laboratory training.

The simplest version of objective testing is the observation of user behavior supported by audiovisual recording [41]. However, the research data collected by so-called objective observers is not free from reliability issues. Apart from the relatively high organizational ease of conducting such sessions it is difficult to find any other advantages, except for observations conducted by specialists/experts in a given field, as presented in [42].

The most reliable and objective usability tests are based on analyzing the user’s recorded biomedical signals [24]. It seems that motion tracking [43,44], eye tracking [45], heart rate [46], EEG signals [47], speech analysis, etc. are the most suitable for usability studies of VR applications. Due to the complexity of the acquired signals, ML/AI methods are often used to interpret the user’s emotional state [48,49]. They are undoubtedly endowed with higher reliability, however, it is difficult to apply them on a mass scale. Therefore, they are rather crucial for conducting basic research on new UI for VR.

## 3. VR Design

Since the concept of VR is based on complete immersion in a totally different environment, there are some elements that one must consider during the design process. This section lists the differences between VR and other types of applications and summarises some good practices for VR designing.

One of the most significant aspects of VR is the field of view (FOV). FOV refers to the horizontal and vertical angular dimension of the display [50]. The user’s immersion depends strongly on FOV, because the wider the FOV, the deeper the experience is. There are two types of FOV. Monocular FOV, which refers to one eye FOV, is between 170°–175° and consists of the angle from the pupil towards the nose (usually 60°–65°) and the view from pupil towards the side of the head (usually 100°–110°). Binocular FOV, the combination of the two monocular fields of view, provides humans with a viewable area of 200°–220° [51]. Where the two monocular fields of view overlap, there is the stereoscopic binocular field of view, about 114°, where one is able to perceive things in 3D [52]. While a wider FOV is essential for immersion, the stereoscopic binocular FOV is where most of the action happens both in a natural and virtual environment.

Thus, while planning object placement and interaction design, VR designers should consider the user’s point of view. For example, it is very important to fit UI within the user’s field of view and keep the focus on the main action not to distract them and not to break the immersion. What is more, the immersive content should be placed in an optimal zone (distance between the object and the user is between 1.25 m and 5 m), taking into consideration the user’s position (sitting, reclining, standing, or walking), possible adjustment of the content to a different place, and comfort while using the app. Another significant issue of VR is interaction, emphasizing the tactile interaction between the user and the elements of the virtual environment. It is usually handled with different kinds of controllers, haptic gloves, and increasingly more often with hand tracking, which is the most intuitive and natural [53,54]. However, the lack of physical feeling of touching influences the realism and precision of interaction. Thus, different kinds of haptic solutions like force feedback gloves [55] or full-body haptic feedback [56] are frequently used in VR solutions. The other approach allows one to sense virtual elements and manipulate them using a simulator equipped with real components [57]. When designing gesture–controller interaction, it is essential to avoid muscle fatigue. Such a phenomenon can accumulate when users are expected to keep an extremity in a particular position throughout an experience or require the user to make air tap gestures over a long period repeatedly. This goal can be achieved by incorporating short breaks or offering a mixture of gestures and speech input to interact with the app. It is also vital to arrange the interactive elements properly by putting them in the correct height and vicinity for the specific user. Additionally, it is vital to avoid interaction that forces the user to make potentially dangerous movements, as people lose track of the natural world when they are in VR [58]. If the execution of a movement becomes difficult or hazardous, one can take advantage of the speech interface. This intuitive approach reduces time, minimizes effort and cognitive load, and at the same time is more efficient [59].

Suppose the object one has to interact with is far away from the user. In that case, it is essential to add the possibility of conveniently moving around in VR. However, the ability to navigate VR is usually restricted by the system and position tracking of the headset. Consequently, normal movement is severely restricted in VR. Furthermore, numerous studies have proven that navigating a virtual environment influences the sense of presence experienced [60]. One of the solutions is adapting standard 2D environment controllers like joysticks, keyboards, or a mouse into VR. What is more, moving in a VR environment may cause a strong feeling of nausea. There are two reasons: first, in real life, one would be looking towards the direction of traveling most of the time. Therefore, using a controller to move generates a conflict in the sensory system, which causes simulation sickness. Secondly, this method often produces many changes in speed or acceleration, which is another factor of simulation sickness. Therefore, the most manageable solution is to use the user’s head direction to travel at a constant speed. Additionally, the users should be able to indicate the start or stop by pressing a button. Another approach to move around in VR is teleporting. Applying this method, the user can move from one spot to another and by selecting the target location, the user is transferred there immediately. Target locations can be predefined, and users can either look at a spot or point at one with the controller to indicate it [61]. Currently, external devices supporting/stimulating movement in VR are used, such as a treadmill or a sphere. However, alongside modern, original design solutions, applying typical HCI guidelines known as the 8 Golden Rules of Interface Design by Ben Shneiderman [62] (strive for consistency, enable frequent users to use shortcuts, offer informative feedback, design dialogue to lead to closure, offer simple error handling, enable easy reversal of actions, support internal locus of control, and reduce short-term memory load) can be indispensable when creating a VR environment.

## 4. Methods and Analysis

### 4.1. Study Design

The application dedicated to user interface usability testing was prepared using Unity platform. Since the research is in its early stages, we decided to conduct the testing for an extremely simple user interface. Thus, the proposed analysis should be treated as a pilot for future research of complex navigation systems. The studied interface contains only two buttons—YES and NO. The basis of interaction is randomly generated questions and the difficulty level was tailored so as to not pose a challenge for adults and generate stress. The first type of task comprises mathematical operations like addition and multiplication, the second one includes reading time from a clock face, the third and the fourth one require the user to recognise simple geometric shapes and colours accordingly. Each variation of the test task has randomly generated content to achieve unrepeatability of experience. Such an approach allows the same user to be tested with the ability to monitor the real level of engagement in the experiment. The application registers objective digital parameters of the experience: time, undertaken interactions, head and hands movement tracking, as well as speech and EEG signals. The data is recorded at a frequency of approximately 100 Hz as a CSV file. The environment has been selected to not distract the test users, while at the same time providing a high immersion level.

The design of the VR app allowed us to check the usability of several elements/factors that may significantly influence user perception of the application. It contained the following issues:

- issue #1—interface board in the wrong distance from user (too close, too far away);
- issue #2—swapped places of answer buttons;
- issue #3—answers without button (just letters without any frame);
- issue #4—answer buttons out of field of view (also connected with arm fatigue);
- issue #5—haptic inconsistency;
- issue #6—sound inconsistency.

**Figure 2:**
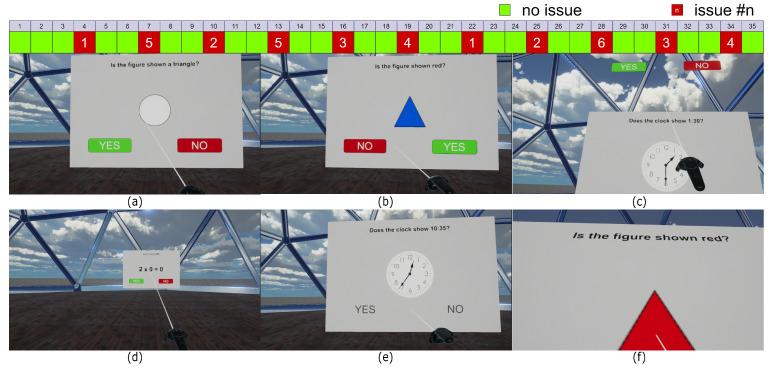
The experiment sequence and sample scenes from the VR application: (**a**) layout is arranged adequately, (**b**) inconsistency—colors of the buttons are changed, (**c**) buttons are located too high, (**d**) elements of the layout are positioned too far away, (**e**) the buttons are replaced by lettering, (**f**) elements of the layout are positioned too close.

The mistakes were included separately in the questions in order to check their influence independently. Moreover, questions with interface errors were always separated from each other by two correct ones. Sample scenes from the VR application are presented in Figure 2.

### 4.2. Study Settings

For the purpose of this research, objective features were acquired using HMD built-in head and hands tracking, EEG sensor and video recording, taking into consideration task performance and speed as well, which are all briefly described in the following section.

#### 4.2.1. HMD Built-In Head and Hands Tracking

Inertial sensors such as accelerometer and gyroscope are commonly used in human activity recognition [63]. In HTC Vive set, spatial localization is possible using two IR base stations, which beam signals to the headset and controllers. The 3-axis accelerometer (ACC) is a device used to measure linear acceleration along three axes. A gyroscope is used for measuring orientation and angular velocity. For the purpose of this research we used accelerometer and gyroscope built in HTC Vive Headset [64] to track the displacement of one’s head and hand from the original position, which provides context information about the physical activity of its user. In HTC Vive set, spatial localization is possible using two IR base stations, which beam signals to the headset and controllers. Since an essential assumption of usability of the VR application is the lack of excessive and disruptive hands and head movements, we decided to track this activity to verify how different their trajectories are in particular tasks. The ACC and gyroscope signal is used to analyze the trajectory of head and hand movement to recognize the deviations and irregularities in the performance of the task. The data is provided by Unity.

**Figure 3:**
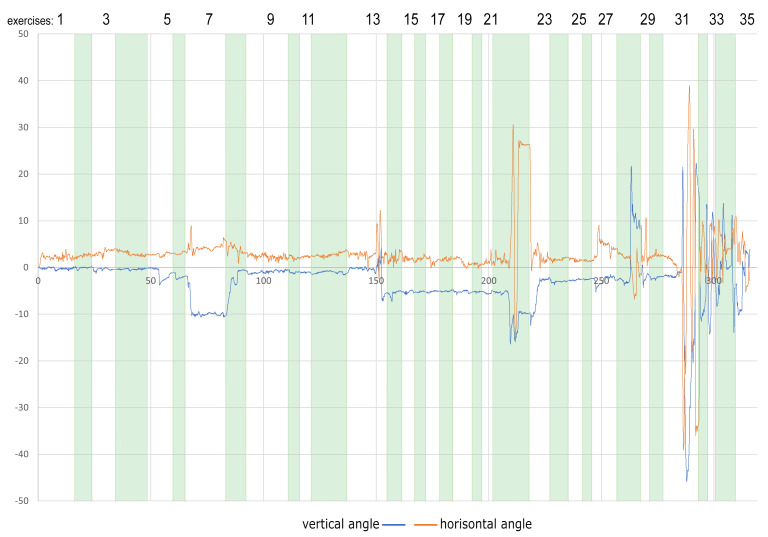
An example of X and Y-axis rotation: head movements of 48-year-old woman during the whole session divided into separate exercises.

Figure 3 presents an example of X and Y-axis rotation: head movements of 48-year-old women during the whole session divided into separate exercises. As one can easily observe, the first six tasks were performed without significant head movements. The slight movement appears during the 7th task (issue #4). Additionally, performing this task increased the time needed to complete it compared to the previous ones. The next increased mobility occurred around tasks 13 (issue #1), 22 (issue #4), and 28 (issue #4). In the case of tasks 22 and 28, the time needed to complete the task is extended as well. The greatest excitement begins from task 31 (issue #1), slowly decreasing from 32 (no issue), but does not return to its initial level.

**Figure 4:**
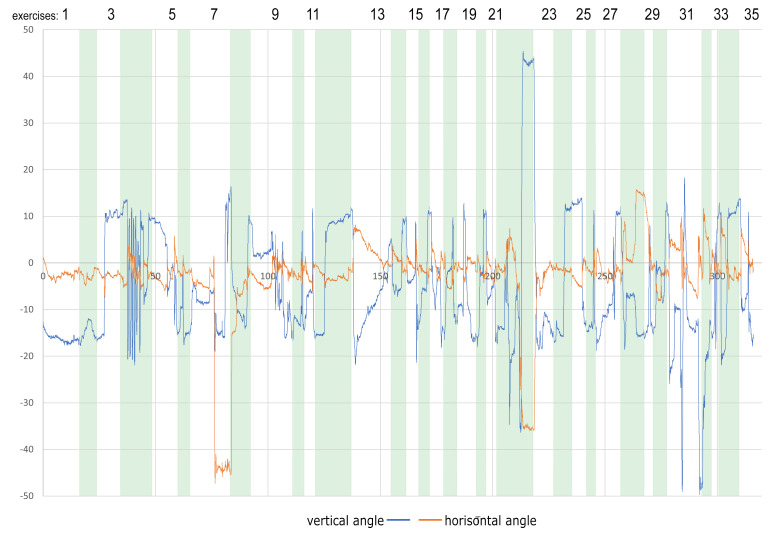
An example of X and Y-axis rotation: hand movements of 48-year-old woman during the whole session divided into separate exercises.

**Figure 5:**
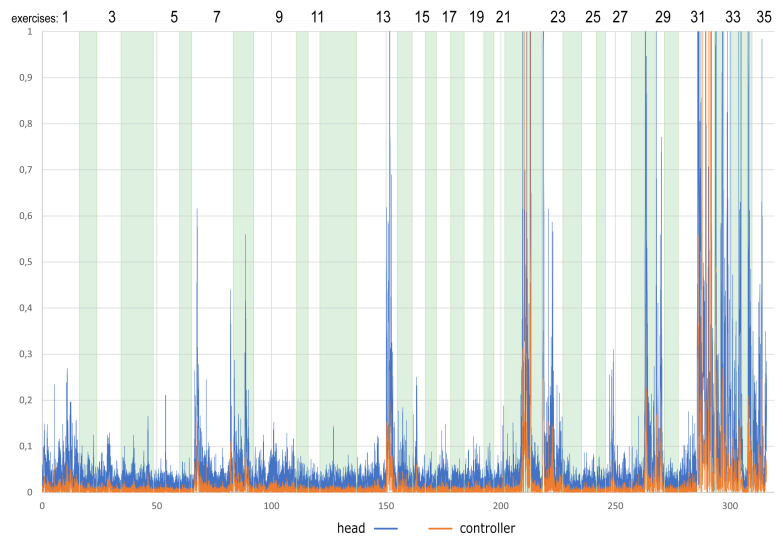
An example of absolute values of the normalized [0–1] angular acceleration: hand and head movements of 48-year-old woman during the whole session divided into separate exercises.

While analyzing Figure 4, one can quickly notice slight hand movements (about 10–20 degrees) in vertical directions throughout the whole session. These hand movements are negligible and will not be taken into account. Significant movements occurred while the user performed tasks 7, 22, and 31, and all of them are connected with issue #4. Similar phenomena can be noticed when analyzing the chart presenting absolute values of the normalized [0–1] angular acceleration of hand and head movements (see Figure 5).

#### 4.2.2. Electroencephalography

EEG is a method to monitor the electrical activity of the brain. The relationship between brain activity and emotions was repeatedly proven. There are two main areas of the brain correlated with emotional states, namely the amygdala and the frontal lobe. For example, it has been found that the amygdala is the biological basis of emotions that store fear and anxiety. Additionally, studies have shown that the frontal scalp stores more emotional activation than other brain regions such as the temporal, parietal, and occipital [65]. However, an EEG headset may be inconvenient since electrodes are placed along the scalp, especially when wearing a VR headset. Thus, for the purpose of this project, we used the Looxid Link device to perceive, evaluate, and monitor the affective state of the user while testing the VR app [66].

**Figure 6:**
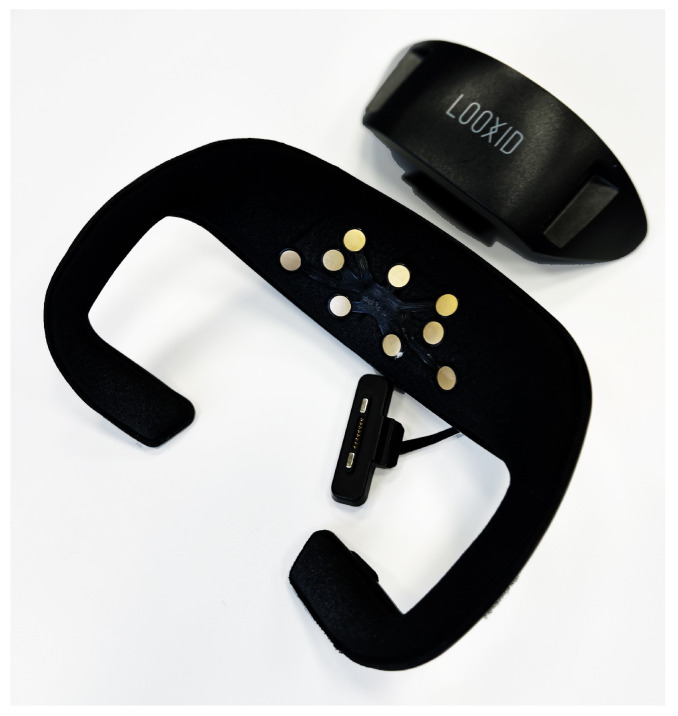
Looxid Link Package for VIVE Pro/VIVE Pro Eye [67].

Looxid Link is an EEG system (see Figure 6) equipped with gold-plated sensors to detect brainwave signals from the prefrontal area. Signals that arise from brain activity are streamed at 500 samples per second to the computer. Using Looxid Link, it is possible to monitor electroencephalographic signals and consequently identify the user’s affect such as attention, relaxation, and brain balance, as well as fundamental characteristics of brainwaves including delta, theta, alpha, beta, and gamma per 100 ms. The signals and the VR content that the user is experiencing can be synchronized on a time basis. Thus, it is easy to connect a specific emotional state with a particular event in the VR experience.

**Figure 7:**
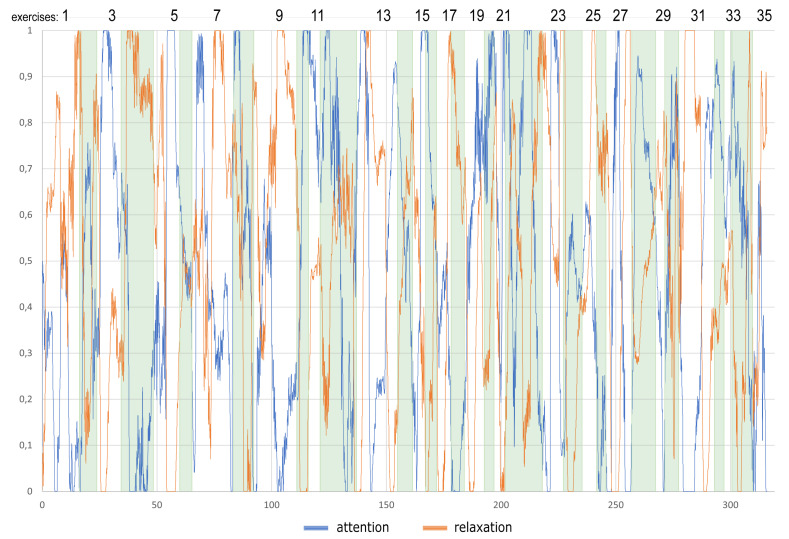
Brainwave recordings interpreted by Looxid Link into two stages (attention and relaxation) of 48 year old women during the whole session divided into separate exercises.

Figure 7 presents an example of brainwave recordings interpreted by Looxid Link as two states (attention and relaxation) of 48-year-old women during the whole session divided into separate exercises. A sudden increase of attention at the beginning of the activity is observed for the following tasks: 3 (no issue), 7 (issue #4), 8 (no issue), 12 (issue #2), 13 (issue #1), 22 (issue #3), 28 (issue #3), 32 (no issue), and 34 (issue #6). A sudden increase of attention while task realisation is observed for the following: 5 (no issue), 13 (issue #4), 15 (no issue), 19 (issue #3), 21 (no issue), 22 (issue #3), and 23 (no issue).

#### 4.2.3. Video Analysis

All usability testing sessions were recorded using a Sony HDR-TD20VE 3D camera to be subsequently analyzed in great detail. It was proven that facial expressions provide high accuracy on what users are feeling in relation to what they are saying [68]. Thus, video recording of the user’s actions and facial expressions contribute to a more detailed analysis of the product usability [24]. However, in the case of VR application testing, where HMD covers almost the whole face of the sample, it is impossible to analyze emotions from the mimicry.

Speech signal analysis was conducted according to our previous work [49,69]. A pool of descriptors commonly utilized for emotional speech recognition such as fundamental frequency (F0), speech energy, and Mel Frequency Cepstral Coefficients (MFCC) was used as input features. To be consistent with the results returned by Looxid Link, we consider two classes: increased activity (a task with the issue) and neutral speech (no issue).

**Figure 8:**
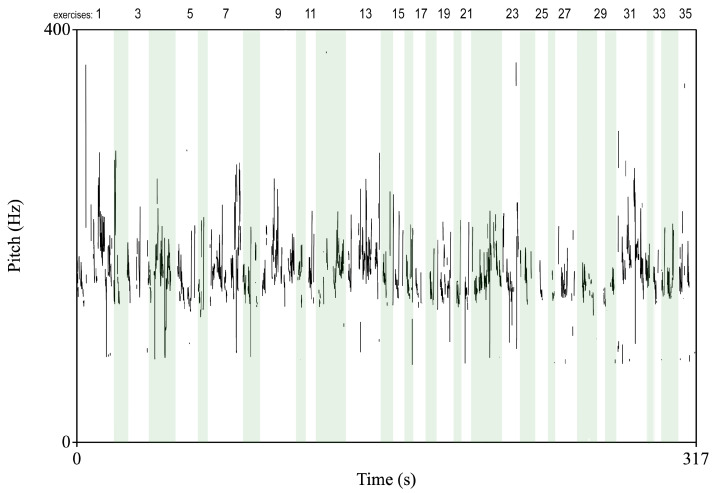
An example of pitch extracted from speech sample of 48-year-old women during the whole session divided into separate exercises.

Figure 8 presents pitch (the perceptual correlate of fundamental frequency) extracted from speech samples of 48-year-old women during the whole session divided into separate exercises. The analysis was carried out using PRAAT [70]. In general, the user’s speech is unvarying and monotonous. The most significant changes in pitch are observed for tasks 4 (issue #2), 7 (issue #4), 13 (issue #1), and 31 (issue #4).

#### 4.2.4. Task Performance

**Figure 9:**
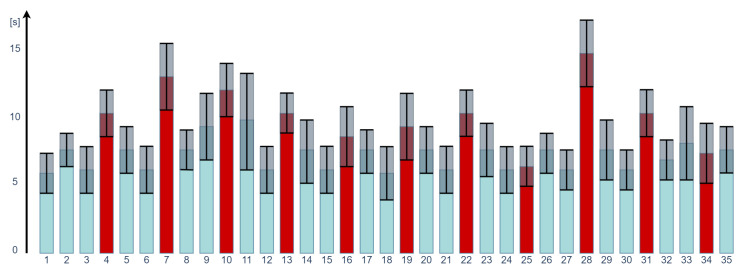
The average speed of tasks performance by all participants. In blue-no issue tasks, red-tasked with the issue.

The ability of users to complete top tasks is the most effective measure when testing the usability of websites [71]. It can be measured as speed (how long it takes to complete the task) and correctness/precision (whether the task is completed correctly or not). Thus, we validated the effectiveness of both metrics in the case of VR usability testing. Figure 9 presents the average speed of tasks performance by all participants.

As one can easily observe in most cases, the average time taken to complete the no-issue tasks is shorter than in the case of tasks with issues. As one can easily observe in most cases, the average time taken to complete the no-issue tasks is shorter than in the case of tasks with issues. The only exception are tasks #25 (haptic and sound inconsistency) and #34 (sound inconsistency).

The correctness of the performance of the task did not affect the results at all—over 98% of the answers were correct (the tasks were elementary). Therefore, we did not include this parameter in the feature vector.

#### 4.2.5. User Observation Analysis

In order not to disturb the user during the session, the observation was performed based on video recordings. The camera was set in front of the monitor to capture the preview of what the user sees in VR (the observer knows what task the user is facing in a particular moment). The professional observer took notes regarding user’s behavior and reactions to encountered UI errors. After testing the application and filling in the questionnaire, there was an additional in-depth interview focused on the user’s feelings and impressions. After analysis of the observation phase of sample #3, the following elements were pointed out: task 4 (issue #2)—the user was confused, instinctively wanted to press YES in the “correct” position, needed more time to find the new order of the buttons; task 7 (issue #4)—significant head movement occured, the user was surprised, there were no extra controller movements, the user used the controller just to select answers; task 13 (issue #1)—the user could not easily read complete information, needed additional head movements to read and choose the answer; task 19 (issue #3)—the user was surprised; task 22 (issue #4)—the user was confused, did not know what to do, needed more time to familiarize with the changed UI, significant hand and head movements occured; task 28 (issue #4)—significant hand and head movements occured; task 31 (issue #1)—the user did not see what was on the board, it was challenging to read, needed more time to familiarize with different UI, significant hand and head movements occured as well.

### 4.3. Procedure

**Figure 10:**
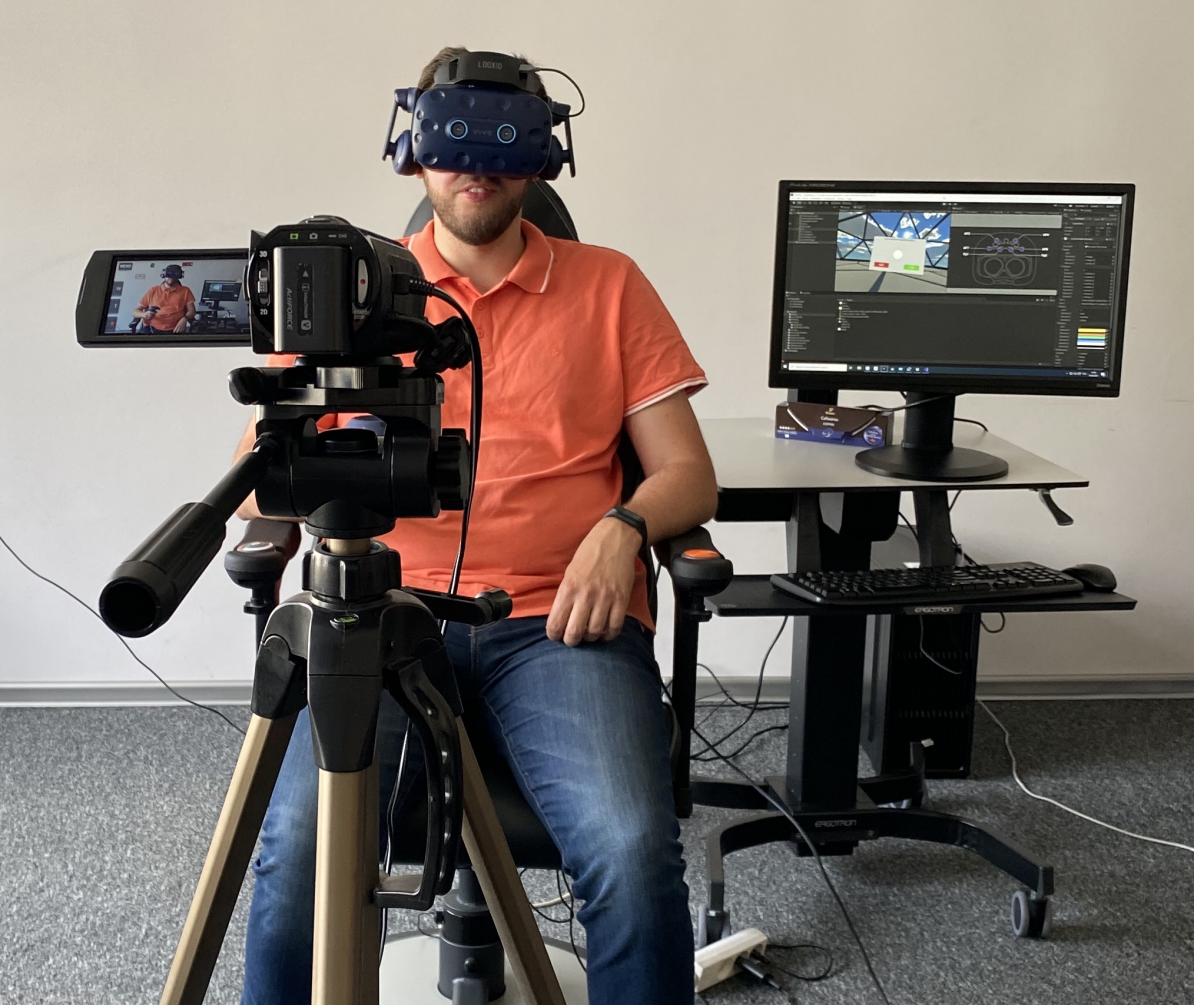
A snapshot of a subject during a VR application usability testing session.

The pilot study included fourteen volunteers (samples). They were informed about the purpose of the study. The procedure of the experiment was explained to them step by step. Volunteers were told that they had the right to withdraw their consent to participate in the study at any moment. At the beginning of the experiment, the operator presented the subject with the standard protocol of the investigation. The protocol includes information on the aim of the study, the procedure of sensor installation, and the time of VR immersion. All samples were free to ask any questions they deemed necessary. The order of questions for the given sample was random. The sequence of the actions was the same in each session: information to the sample, sensors and VR headset mounting, immersion, sensor, and VR headset unmounting, and a questionnaire and a short interview. A snapshot of a subject during the VR application usability testing session is presented in Figure 10. Participant characteristics are presented in Table 1. On average, samples were 40.2 years old (median = 40, std = 10.8, range = 26–62) and the group comprised nine men (mean = 42.5, median = 43, std = 11.12, range = 31–62) and five women (mean = 36, median = 37, std = 8.87, range = 26–48).

### 4.4. Automatic Usability Testing

**Figure 11:**
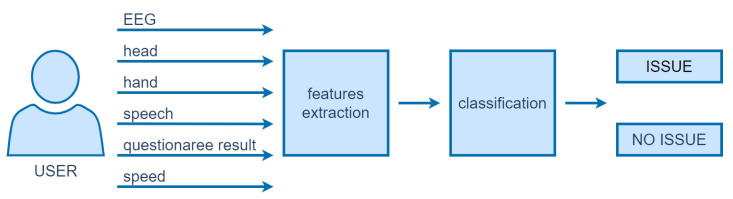
Main components of the proposed system.

In this section we present the main components of the proposed system. Studies have been carried out according to the algorithm shown in Figure 11. The main steps are described in the following section.

#### 4.4.1. Features Extraction

As it is presented in Figure 11, a set of features was extracted from each captured data set (see Table 2). Head and hand movements are described using mean, median, standard deviation (std), maximum, and minimum of X and Y-axis rotation values (20 features in total). In the case of EEG, data provided by Looxid Link (level of attention and relaxation) in the form of the same statistical features is taken into consideration (10 features in total). For speech signal MFCC (mean values of 13 MFCC coefficients), F0 (mean, median, std, maximum and minimum), and energy (mean, median, std, maximum and minimum) were extracted—23 in total. It has to be underlined that these features were extracted for each task performed by a particular user separately.

#### 4.4.2. Classification

The final step of the proposed method is classification, which aims to assign input data to a specific category k (in this case: issue and no issue). In this work, we apply different machine learning methods to the proposed combination of datasets to compare their performances, based on the recognition rates. The verification of efficiency of feature subsets is carried out using several types of classifiers such as k-nearest neighbors algorithm (k-NN), support-vector machines (SVM), Multilayer Perceptron (MLP), and Random Forest (RF) using Weka [72], with 10-fold cross-validation. This approach allows the evaluation of the efficacy of particular features set and determines the most efficient ones. In the course of the research, the parameters for each classifier were identified and selected to achieve the highest recognition results. The number of samples in both sets (*issue, no-issue*) was normalized.

## 5. Results Discussion

### 5.1. Automatic Usability Testing Results

It is clearly visible (see Table 3) that the best results are achieved for the subsets containing speech signal features (68.51% using SVM). We suspect that this may be related to silence during issue-free tasks. The lowest results are collected in hand movements (64.94% using RF), which is noticeable for all types of classifiers. Analyzing results retrieved from different patterns, a significant recognition rate improvement when using the RF classifier can be observed in most cases. It is very evident, especially for hand movements. Only in the case of speech signal does SVM give better results than RF.

**Figure 12:**
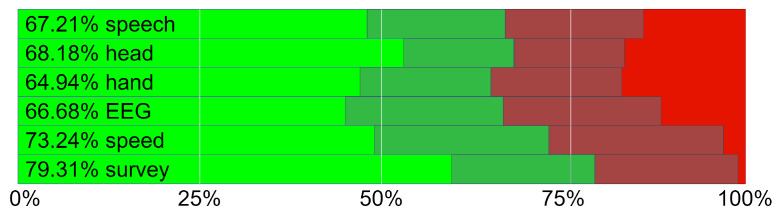
Mean values of factors with 95% confidence interval of the T distribution.

The overall accuracy using all features as an input vector (COMBO) can be observed compared to one source-feature set. Additionally, in this case, the best results were achieved with RF (71.75%). On the other hand, the lowest increase of results is observed for the k-NN algorithm (67.21%). As expected, the effectiveness of classifiers whose testing and training sets comprised features gathered from different sources supported by the questionnaire results and speed of task performance (COMBO+Q+S) is much better than those operating on one particular feature set and even better than COMBO and COMBO+Q (different sources supported by the questionnaire results). The number of features by combining each source set increased the quality of the classification. The best results obtained using RF are as high as 84.23%. Mean values obtained using RF of factors with 95% confidence interval of the T distribution was presented in Figure 12.

### 5.2. Usability Questionnaire Results

Table 4 presents the summary of the questionnaire filled in by all participants. It contains the average outcome of each answer and its standard deviation. The testers agreed on the fact that the application was easy to use even though there were some inconsistencies and UI errors. Moreover, the typical colours of the buttons (green for YES and red for NO) helped users to select the answer in a shorter time. The change in sound was confusing for almost all participants. More significant differences among the users were noticed in other tasks. Interestingly, the opinions diverged in issue #2, as half of respondents found the application too inconsistent whereas the other half presented the opposite attitude. The questionnaire confirmed the observations—50% of participants used the UI located too far without any trouble while 36% did not feel comfortable to read and perform this task. Taking into consideration the button design, the majority of users found it easier to select answers in the form of a framed button. 58% thought the user interface placed too close was uncomfortable to use. It might be connected with the necessity of unnatural head movement and additional effort to find and click the answer button. Placing the answer buttons out of the field of view was assessed as an element of moderate usability, which might be linked with user expectations connected with VR experience—some head movement is anticipated and desired. It is important to notice that users get used to UI arrangement and any changes in it may lead to confusion and discomfort in use.

### 5.3. Usability Observation Evaluation

After analysis of the observation phase, the following conclusions might be drawn:

- The distance between user and UI board does matter. 62% of users were confused and rather taken aback when the UI was too close; some of them nervously looked around to read and choose the chosen answer; selection of the correct button was challenging for them as well. 29% of test participants had problems seeing clearly and reading when the UI was too far. Interestingly, older participants and those with hyperopia did not find this error problematic.
- Users get used to UI arrangement (in this case button arrangement). When the answer buttons (YES/NO) were swapped, 21% of users were confused and unsure if they had selected the correct answer. The design of answer buttons with frame and colour (YES—green, NO—red) helped users to find the desired option. Without buttons, 36% of users did not know where they should exactly press. When the buttons were out of field of view, all users needed more time to find out “new arrangement”, so they used more time for such questions (statistically over twice as long). However, the users adapted to buttons outside the board and there was less confusion in the next questions with such UI error.
- Controller vibrations do not play a key role in VR experience. 57% of participants did not notice any changes in controller vibrations, and the majority of them did not feel any haptics.
- Sound is a valuable element of UI and supports giving user feedback as well. When the sound was changed a few users were confused and unsure whether they had selected the correct answer.

## 6. Conclusions

The assumptions made about users’ cognition and signal analysis performed based on them were not fully satisfactory. While 84.23% maximum recognition rate is not high enough to consider it a valid and proven automatic usability testing method, it is definitely enough to grant further exploration. It can be considered as an interesting starting point for further studies on automated usability testing of VR user interfaces. The research conducted on a relatively small sample of users has yielded a number of experiences and suggestions for further pursuit of best practices. The level of the obtained results is so satisfying that the team sees great potential in further work on the topic. We believe that refining and specifying research tools and procedures will significantly increase their efficacy and the developed methodology will find its application in professional screening and/or mass production research.

Still, many variables are highly user-dependent. Responses and comments vary depending on the individual and automation can provide above 84% of accuracy of recognition, which is a promising value considering relieving the role of an observer in case of mass tests. As can be observed in the results, one feature is not enough and a combination of features gives better results. Thus, further research should focus on extracting more sophisticated features from already obtained signals (e.g. instead of using stock features of Looxid Link, consider using raw EEG signal) or collecting data from additional sources like eye tracking, infrared thermal imaging, galvanic skin response, motion capture, etc. However, we must remain aware of the fact that collecting such data requires additional external equipment, which might influence the comfort during the experience and consequently misrepresent the usability test results. The questionnaire at the final part of the testing process might be changed into a more immediate one, thus increasing its relevance. While asking the users for a direct, immediate response after each critical event would definitely increase response accuracy, such an approach might introduce an undesired disruption of the VR experience. An attempt should be made to separate the time intervals in which the user is undertaking interaction with the UI and the time necessary to perform the actual VR task.

## Figures and Tables

**Table 1:** Participant characteristics.

Partic.	#1	#2	#3	#4	#5	#6	#7	#8	#9	#10	#11	#12	#13	#14
**Sex**	M	M	F	F	F	M	M	F	M	M	M	M	F	M
**Age**	62	50	48	43	26	32	57	26	44	31	33	31	37	43

**Table 2:** Features extracted from each captured data set.

Head movements	Y-axis rotation: mean, median, std, max and min
	X-axis rotation: mean, median, std, max and min
Hand movements	Y-axis rotation: mean, median, std, max and min
	X-axis rotation: mean, median, std, max and min
EEG	Level of attention: mean, median, std, max and min
	Level of relaxation: mean, median, std, max and min
Speech signal	MFCC: mean values of $MFCC_{1}-MFCC_{13}$
	F0: mean, median, std, max and min
	Energy: mean, median, std, max and min

**Table 3:** Classification performances (in %) of different feature representations for the set of two classes: issue and no_issue task. Numbers in bold highlight the maximum classification rates achieved in each column. COMBO refers to all extracted parameters in one feature-vector. COMBO+Q refers to all extracted parameters supplemented the score from the questionnaire obtained for a particular issue in one feature vector. S means speed in task performance.

	k-NN	SVM	RF	MLP
speech	64.29	**68.51**	67.21	61.69
head	66.23	66.23	**68.18**	66.68
hand	55.84	58.77	**64.94**	61.69
EEG	66.56	64.94	**66.68**	65.58
COMBO	67.21	68.51	**71.75**	70.78
COMBO+Q	73.70	75.97	**76.95**	76.30
COMBO+Q+S	79.8	80.82	**84.23**	82.20

**Table 4:** Usability Questionnaire for users.

Questions	Mean	STD
#1 I thought the application was easy to use.	4.6	0.6
#2 I thought there was too much inconsistency in this application.	3.1	1.4
#3 I found the buttons that were too low or too high very cumbersome to use.	3.5	1.1
#4 I felt very uncomfortable/unconfident when the buttons were out of field of view.	2.6	1.2
#5 I thought the user interface placed too close was uncomfortable to use.	3.9	1.3
#6 I thought the user interface placed too far was difficult to use.	2.8	1.4
#7 I found the colour of buttons (green for YES, red for NO) helpful to select wanted answer.	4.5	0.6
#8 I needed more time to select the answer when the buttons were in opposite colours (YES—red, NO—green).	2.8	1.4
#9 I found it surprising when the sound confirming selection was different than previously.	4.4	0.6
#10 I found it easier when the answers to select when in button-form (buttons in a frame with colour background).	3.6	1.4
